# Proceedings of the Montgomery Co. Society, April, 1858

**Published:** 1858-07

**Authors:** 


*Proceedings of the Montgomery Co. Society, April,* 1858. Reported by C. M'DERMONT, M. D., Dayton, Ohio.

*Dr. Carey* read a paper on the "Toxical action of Veratrum Viride upon Dogs."

Dr. Carey said, that the time allotted for the consideration of *veratrum viride* had been too brief for him to prepare anything like an elaborate report on the subject. He had made some experiments with the article upon dogs, which were designed to illustrate the toxical effects of the agent, and would submit them for the consideration of the Society this evening.

The *first case* to which he administered the Veratrum—Tilden's Fluid Extract—was a young spaniel, laboring under influenza. His respiration was hurried, with pulse 158 per minute. The dose was six drops. The dog vomited in thirty minutes, which act was repeated in an hour. In 2½ hours his pulse was 100 per minute.

*Case* 2.—A small cur dog took 20 drops; pulse 106; respiration 1 to 4 pulsations. In eight minutes evidenced nausea, in ten vomited, which was repeated at short intervals for half an hour; in fifteen minutes, pulse 70; in thirty, pulse 40; in fifty, pulse 32; in 1¼ hours, pulse 40; in 1½, pulse 68; in 1¾, 72; in 2½, 60. In thirty minutes after taking veratrum, animal purged and staggered in walking, in sixty minutes could not stand. This prostration continued for three hours. In fifteen hours animal felt well and partook of food with avidity.

*Case* 3.—On the subsequent day, gave same animal fifteen drops veratrum and half a grain of morphia—pulse 84; in half an hour, pulse 96, no nausea; gave fifteen drops more veratrum, and in an hour, pulse 86; gave thirty drops additional, without influencing in the least perceptible degree, either the heart or stomach.

*No.* 4.—Next day, gave same dog thirty drops veratrum and sixty drops laudanum, with pulse 100. In thirty minutes, pulse 80; in sixty, pulse 72, irregular; in two hours, 60; in four hours, 72. No nausea, respiration irregular and sighing.

*No.* 5.—Next day gave to above animal, with pulse 136, one hundred drops veratrum. In ten minutes, pulse 72; in fifteen minutes exceedingly irregular and intermitting; in twenty fluttering, with an intermission of about one-half of the time; in twenty-one minutes, heart ceased to pulsate, and in twenty-three minutes ceased to respire. Dog vomited in eight minutes, urinated several times, did not purge, respiration hurried, and sighing after ten minutes, finally became irregular and slow until it ceased entirely. There was no stertor, nor spasmodic motion of the voluntary muscular system.

*No.* 6.—Gave a medium sized dog twelve drops of veratrum, with pulse 120. In sixteen minutes, pulse 100; in thirty minutes, pulse 76, in which condition they remained for three hours, and rallied. Vomited in ten minutes, and continued at short intervals for half an hour; in thirty minutes purged. There was no prostration of the voluntary muscular system in this case, nor depression of spirits.

*No.* 7.—Subsequent day, gave to this dog seventy drops veratrum, with one-third grain of morphine, pulse 90. In two minutes respiration hurried and panting, urinated frequently, nausea, and in six minutes vomited; in fifteen minutes pulse 40, purged; in thirty minutes, pulse 12, regular; can't stand. Nausea still distressing, respiration slow and calm. Gave morphia half a grain and wine half an ounce. No more vomiting; in forty minutes pulse 20, in sixty minutes pulse 48; respiration 12, in which condition animal remained for four days. Next morning felt well, pulse 112, and partook of food with relish.

*No.* 8.—Threw into the rectum of same dog, on next day, 100 drops of veratrum and half grain of morphine. In two minutes evacuated contents of rectum; in seven minutes commenced panting; in thirteen minutes, great nausea, pulse 30; in fourteen minutes, vomited; fifteen minutes, pulse 32, irregular; twenty-five minutes, pulse 160, respirations 80; can't stand; can't count pulse. Took morphine half grain, and wine half an ounce; sixty minutes pulse fluttering, still can't be counted; passed a current of electro-magnetism through the chest behind the fore legs; in seventy minutes, pulse more regular; seventy-five minutes, slower and still more uniform; respiratious slow and deep, animal somnolent. In one humdred and twenty minutes, pulse 160, from which condition the dog gradually recovered. This experiment was repeated with similiar results.

### SUMMARY.

| Case. | Dose, | No. pulse. | Time of vomiting. | Minimum pulsation. | Time of minimum pulsation. | Reduction. | Anodyne. |
|---|---|---|---|---|---|---|---|
| 1 | 6 | 158 | 30 min's | 100 | 2½ hours | 58 | |
| 2 | 20 | 106 | 10 " | 32 | 50 minutes | 78 | |
| 3 | 60 | 84 | — | 80 | 1¼ hours | 4 | ½ gr, mor. |
| 4 | 30 | 100 | — | 60 | 2 " | 40 | 60 d's lau. |
| 5 | 100 | 136 | 8 min's | 72 | 10 minutes | 64 | |
| 6 | 12 | 120 | 10 " | 76 | 30 " | 46 | |
| 7 | 70 | 90 | 6 " | 12 | 30 " | 78 | ⅓ gr, mor. |
| 8 | 100 rec'm | 100 | 14 " | 22 | 15 " | 78 | ½ " " |

From the foregoing it will appear, that *veratum viride* when administered in large doses to dogs, is a powerful narcotic poison, capable of destroying life. It produces its toxical effects, first upon the ganglionic, and finally implicates to a limited extent, the cerebro-spinal system of nerves. When given alone, it greatly reduced the force and frequency of the heart's action. When combined with morphine or laudanum, this action is modified. The greatest reduction of the heart in the experiments was 78 beats. This obtained in three instances, viz: No. 2, where 20 drops were given alone, and Nos. 7 and 8, where 70 and 100 drops were combined with ½ grain of morphine, and respectively taken per mouth and anus.

The shortest time occupied in reducing the heart to its minimum action was ten minuter. This took place at the rate of 6 $\frac{7}{16}$ beats per minute.

When portion under 20 drops were administered, the pulsations of the heart were quite regular and the diminution of action uniform, until the minimum was attained; but during the rallying stage, the action was in all cases irregular and intermitting.

The longest time occupied in inducing vomiting was fourteen minutes, and that was when the article was thrown into the rectum. In two cases, it occourred in ten minutes, in one six, and another eight. In all it was protracted and the nausea distressing. In the case that proved fatal, death commenced at the heart; this organ ceasing to pulsate two minutes before the lungs ceased their function.

The influenoe which opiates exerted in these cases is of great interest. In No. 3, 60 drops of veratrum, with ½ grain of morphinae, did not affect the circulation and stomach. In No. 4, 30 drops of veratrum and double the quantity of laudaunm reduced the heart forty beats in two hours, but did not produce nausea. In No. 7, 70 drops of veratrum, with ⅓ grain of morphine, produced no greater effects upon the animal than 20 drops did in case 2. So, too, of No. 8, where 100 drop were thrown into the rectum with ½ grain of morphine. As to the facts in these cases, there can be no question; but they require additional confirmation to establish the antidotal powers of opiates over veratrum viride.

*Dr Denise* complimented Dr. Carey on the result of his experiments. These were highly valuable in a scientific point of view. He hoped his friend would continue his investigations, as the subject of veratrum viride was at present one of deep and general interest among medical men. He looked upon veratrum as the greatest remedy of the age. He was in the hapit of using it in a great variety of cases, and with the happiest results. Had used it successfully in pneumonia, bronchitis and convulsions. In cases of pneumonia, had used the veratrum alone with marked benefit. Has found it act like a charm in allaying the catarrhal fever of children. He mentioned a case of a child that was subject to attacks of this kind; they were always promptly relieved by the veratrum, which the mother was in the habit of administering at every onset of the disease. He had never kuown it to fail in reducing the heart's action.—It occassionaly produced nausea, but no other unpleasant effect. He did not know how the old doctors got along without it.

*Dr. Brenton,* had not used the veratrum extensively, but from his experience, he was induced to regard it with much favor. He thought it suitable in a great variety of diseased conditions, and especially in diseases of the heart; had given it for three months to a lady who was suffering with hypertrophy of the heart. Prior to its use, her pulse averaged 130 per minute, and she had a bad cough. The veratrum (uncombined) lowered the pulse to 68, and re moved the cough entirely. During this period, he occasionally suspended the medicine, and found that very soon the pulse would rise to its former frequency, and the cough would return, to disappear again on the re-administration of the medicine. Finally, the lady refused to take the veratrum, and the consequence was, that she died soon after, as he had predicted in the event of her discontinuing it. When combined with an opiate, it produces very little nausea. It is not so depressing as antimony, and may be safely used in cases where antimony is inadmissible. Recommended its use in typhoid fever, Had witnessed its good effects in a case of typhus fever. It controlled arterial action without nausea, and according to his observation, it controlled as promptly with, as without opium. He had cured a case of covulsions with it—the convulsions occurred in a young lady; she had *paroxysms almost daily* for two or three years, He gave the veratrum combined with valerian; the convulsions gradually become lighter under the use of the medicine, and soon wholly disappeared.

*Dr. Armor* had listened with interest to the Report of Dr. C.'s experiments. As yet, but few experiments had been made with this agent on the lower animals, and our knowledge of its effect on the human system was confined to the diseased state. It is important to obtain all the facts that can be developed in relation to a subject of this kind. Every fact contains an elment of usefulness, and though considered in its isolated state, in may be worthless, yet when conjoined with other facts of similar nature, it furnishes the material out of which and upon which the truths of science are established.

The experiments detailed contain evidence of one fact, namely, that veratrum tends to produce death at the heart, by virtue of its depressing influence on that organ. This single fact would teach us the propriety of avoiding its use in all diseases which tend to produce death at the heart. In this class we, of course, would include typhus and typhoid fever and all forms of asthenic disease.

In active inflammatory disease, it is very desirable to quell the violence of the heart's action, and in such cases veratrum will do good as an antiphlogistic. It should not be used where there is any local congestion, as it would be apt to increase the stasis of the blood. Its action on the arterial system was not to be doubted, but whether the veratrum acted directly or through the ganglionic nerves of the vascular system, he was not prepared to say.

If there was organic disease of the heart, the veratrum, in his opinion' would be a dangerous remedy. It was powerful for good or evil, and should be used cautiously. He had never witnessed any cumulative effect on the brain or cord, as was met with in the use of digitalis. He had frequently administered it without any perceptible result. Once he kept a patient on the use of it for some days without at all affecting the pulse; and this led him to remark that there might be an error in attributing its inactivity in certain cases to the controlling influence of opium

*Dr Carey's* views of the properties of veratrum had been confirmed and enlarged by his experiments. He would not use the remedy where there was mechanical obstruction to the heart's action, neither would he commend its use in asthenic cases. The controlling influence of opium over the action of veratrum, when administered in combination, was evident from his experiments, and accorded with his experience in practice. A patient who had been using morphia, took the veratrum in full doses without the ordinary effect. He administered it to another patient in combination with laudanum, and the pulse remained unchanged. He had given it, in the same combination to a female who had diarrhea, and was threatened with mammary abcess. She was cured, but the specific action of veratrum was not manifested in her case. He had always found veratrum act as certainly, as efficiently, and as rapidly as any other article of the materia medica. The remedy gave him complete control over the pulse. He could hold it at 40, 50, or 70 as he pleased, and as long as he pleased. Its full action is often unattended with nausea—the depressing effects soon subside. Never noticed any cumulative tendency, except in one case. Uses it freely in inflammatory diseases, in rheumatism, pneumonia, etc. He has found it to allay the constitutional excitement of parturient women, better that anything else. It cures the *weed* like a charm, often producing through the mother anodyne influence on the child.

*Dr. Reeve* thought if veratrum viride had a voice, it would exclaim, "Save me from my friends." Some of the speakers seemed to have laid aside all reason and moderation in their admiration of the wonderful virtues of this drug. We have heard how a baby went promptly to sleep after nursing, because the mother had taken veratrum. This must seem a strange assertion to those who knew, as he did, that all babies are in the habit of falling asleep after nursing, even when no medicine has been administered to the mother. He deprecated the practice of giving veratrum to allay the constitional excitement following labor. He had always been accustomed to see mothers pass safely through this excitement without the aid of veratrum, or indeed of any other medicine. He thought no medication was the best practice in such cases.

*Dr. Carey* explained by saying that he was not in the habit of giving the medicine to all females after their delivery, as was insinuated—but only to those cases where there was high febrile excitement.

*Dr. Reeve* thought the veratrum might be used in some cases with advantage. He was desirous that observation and conclusions should be made with more care. Such crude experiments and unwarrantable deductions would not draw him away from the use of medicines which he had hitherto been accustomed to rely on.

*Dr. T. Brennan*, having never used veratrum, had no prejudices *pro* or *con.*, and therefore considered himself in a better condition to pass judgment upon its merits than were those gentlemen who, in their blind enthusiasm, had been led to attribute to this agent all the good result that follow its use. This *post hoc, ergo propter hoc* style of reasoning had been the grand fallacy of the medical profession in all ages, and is still the most fruitful source of error. One would suppose, from the way some of the gentlemen talk of this new remedy, that their patients would all have died had it not been for its magical effects; and some of its admirers seem utterly at a loss to conceive how any body recovered from sickness prior to its discovery.

He had listened attentively to the report of Dr. C. He had great regard for the Doctor, but his love for scientific truth was greater, and he felt constrained to say that the experiments made upon the innocent dogs had no practical value, and shed no ray of light on the therapeutical properties of veratrum.

He was not prepared to admit that a medicine forced down the throat or up the rectum of a *well* dog would produce the same symptoms that it would do in one of us if we were sick and took it. The dog must be *sick* too before he would admit the completeness of the analogy. He had no faith in this cruel and fatal drugging of dogs. Such experiments might affect the price of sausages, but he was very confident it would add nothing to our stock of scientific knowledge.

His young friend on the right seemed to regard veratrum as a panacea. We are told that mothers keep it on the shelf and give a few drops to the children on the first approaceh of disease, and cure them instantly. If all that is claimed for the remedy is true, there will be no futher need for Doctors. Every old woman can doctor her own household, and we shall find ourselves in the condition of Othello. This would be a sad catastrophe!

The conflicting testimony which the advocates of veratrum have given is rather amusing. He feared it would injure the sale of Norwood's tincture. Some of its friends would use it freely in all sthenic cases, but pronounced it dangerous in asthenic cases; some would use it in asthenic cases, and pronounced it valuable in almost every case; others were opposed to its use in any case. He included himself in the last named class. Common sense would forbid the use of so depressing an agent in asthenic forms of disease; a man in a weak, sinking state is not to be cured by making weaker. To give it in the sthenic state, would only make bad worse—it would convert the case from sthenic. According to his experience, people usually died in the asthenic state, and when he had a patient in the sthenic state, he tried to keep him there. Gentlemen might smile—but he would assure them he had never lost a patient in the sthenic state, and he would venture to say that he had lost as many as some of his neighbors.

His chief difficulty in treating the sick was to combat the downward tendency—to maintain the activity of the vital forces. If the gentlemen would give quinine in pneumonia, and good whisky punch in typhoid, they would lighten the labors of the undertaker. All diseases in this region require to be treated with tonics and stimulants. * * It is strange to what absurdity men's enthusiasm will sometimes lead them.—

One of the brethren had related the case of a young lady who stopped taking the medicine at a time when it was exerting the happiest influence on her diseased heart. The Doctor threatened her with death and a coffin in case she discontinued the medicine. She however, persisted in her refusal, and died soon after. Now, if the veratrum in this instance had so happy and salutary an effect on the lady's heart, as the Doctor says, how very strangely it must have operated on her mind, that she should abandon it at such a time, and in utter disregard of the Doctor's fearful prognosis. As the lady evinced good sense when she applied to the Doctor for relief, are we not warranted in the inference that the veratrum made her crazy? He was sorry friend Carey had not noted whether there was any evidence of mental aberration in the dogs while undergoing the experiments. In conclusion, he would say that veratrum was a humbug, that those who believed in its wonderful efficacy are humbugged, that Norwood, who revived the use of it, is a humbugger, and that the poor patients who have to swallow it are egregiously humbugged.

*Dr. Koogler* thought the veratrum as uncertain as digitalis. He admitted its value in certain cases, but said it was difficult to determine in what particular cases its administration would be beneficial. He did not use it—being unwilling to discard known and reliable remedies for one about which there is so much doubt and contrariety of opinion.

*Dr. Brenton* read an essay on Uterine Hemorrhage, which elicited some discussion.

A case of *delirium tremens* treated successfully by Norwood's Tincture, was reported; the case occurred in the practice of Dr. Clements.

*Dr. Carey*, who had been appointed to prepare an address on the character of the late Dr. Koehne, discharged this duty in a manner which reflected much credit on himself as well as on the memory of the deceased. We suppose the address will be published.

*Drs. Grundy* and *Denise* were appointed Essayists for the next quarterly meeting.

*Dr. Davis* announced the death of D. B. Van Tuyl, M. D., of Indiana, an honorary member of the Society. He also presented a series of resolutions referring in appropriate terms to the eminent qualities of the deceased, and expressing the deep regret which the Society experienced on learning the death of an honored brother.—*Cincinnati Lancet & Obs'r.*

